# A Real‐World Study Characterizing a US Population of Patients Receiving Rituximab With Gemcitabine and Oxaliplatin for Relapsed or Refractory Large B‐Cell Lymphoma

**DOI:** 10.1002/jha2.70351

**Published:** 2026-08-13

**Authors:** L. Elizabeth Budde, Adam J. Olszewski, Bei Hu, Nikesh N. Shah, Connie Lee Batlevi, Shen Yin, Iris To, Michael C. Wei, Martine Joanna Kallemeijn, Lisa Musick, Linda Lundberg, Natasha Shamas, Mark Dixon, Navdeep Pal, Ashwini Shewade, Jason Westin

**Affiliations:** ^1^ Department of Hematology City of Hope National Medical Center Duarte California USA; ^2^ Brown University Providence Rhode Island USA; ^3^ Department of Hematology Atrium Health Levine Cancer Institute Wake Forest School of Medicine Charlotte North Carolina USA; ^4^ Tampa General Hospital Cancer Institute Medical Oncology and Hematology Tampa Florida USA; ^5^ Genentech, Inc. South San Francisco California USA; ^6^ F. Hoffmann‐La Roche Ltd Basel Switzerland; ^7^ Roche Products Limited Welwyn Garden City UK; ^8^ Department of Lymphoma and Myeloma The University of Texas MD Anderson Cancer Center Houston Texas USA

**Keywords:** outcomes, R/R LBCL, real‐world data, R‐GemOx, treatment patterns

## Abstract

**Introduction:**

Data for the use of rituximab, gemcitabine, and oxaliplatin (R‐GemOx) for relapsed/refractory (R/R) large B‐cell lymphoma (LBCL) in the United States are limited. This retrospective observational study characterizes R‐GemOx treatment patterns and outcomes among patients with R/R LBCL from the nationwide, longitudinal Flatiron Health electronic health record‐derived database comprising patient‐level data primarily from US community oncology practices.

**Methods:**

Patients diagnosed with LBCL who initiated treatment with R‐GemOx in the second or later line of therapy between 2011 and 2023, were included. Patient characteristics, treatment patterns, and efficacy outcomes were assessed.

**Results:**

Of 281 patients, most (66.2%) were treated at community centers. Overall response rate was 45.4% (95% confidence interval [CI]: 38.1–52.9); complete response (CR) rate was 22.2% (95% CI: 16.4–28.8). Median progression‐free and overall survival (OS) were 2.8 months (95% CI: 2.6–3.7) and 12.7 months (95% CI: 10.6–17.1), respectively. In patients who received R‐GemOx in the second line, the CR rate was higher and the median OS was longer versus those who received it in later lines (CR rate, 26.3% vs. 17.4%; median OS, 19.6 vs. 10.0 months, respectively).

**Conclusion:**

These data offer a real‐world benchmark for patterns of use and outcomes of R‐GemOx in patients with R/R LBCL in the United States.

**Trial Registration**: The authors have confirmed clinical trial registration is not needed for this submission.

## 1 Introduction

Chemoimmunotherapy is the standard of care for most patients with aggressive large B‐cell lymphoma (LBCL); however, up to approximately 30% of patients with relapsed/refractory (R/R) disease following frontline treatments have poor outcomes [1]. Historically, the treatment of R/R LBCL has been salvage chemotherapy followed by autologous stem cell transplantation (ASCT); however, the prognosis for transplant‐ineligible patients remains poor [2, 3]. Several new therapeutic options have recently been approved for R/R LBCL, including chimeric antigen receptor (CAR) T‐cell therapies [4, 5, 6, 7, 8, 9], CD20 × CD3 bispecific antibodies [10, 11], and targeted agents such as tafasitamab [12], polatuzumab vedotin [13], and loncastuximab tesirine [14]. Despite the availability of newer treatment options, access to these in the community setting, particularly to CAR T‐cell therapies, can be limited due to cost, geographic location, patient fitness, or rapidly progressing disease [15].

Rituximab, gemcitabine, and oxaliplatin (R‐GemOx) is a commonly used, conventional treatment option for patients with R/R diffuse large B‐cell lymphoma (DLBCL), regardless of their transplant eligibility, and is the chosen comparator arm of several phase III R/R LBCL clinical trials, including: POLARGO (NCT04182204) [16], STARGLO (NCT04408638) [17], SUNMO (NCT05171647) [18], LOTIS‐5 (NCT04384484) [19], EPCORE DLBCL‐1 (NCT04628494) [20], EPCORE DLBCL‐4 (NCT06508658) [21], and NIVEAU (NCT03366272) [22]. However, recent real‐world data showing patterns of the use of R‐GemOx, as well as patient characteristics and outcomes, are limited. This lack of data considerably limits the benchmarking of R‐GemOx efficacy in the United States in the ongoing global trials, in turn, further limiting the interpretation of treatment benefit associated with newer therapeutic options being evaluated through multiregional trials.

Using the Flatiron Health Research Database, which primarily includes representation of patients receiving care in community cancer practices in the United States, this study aimed to summarize characteristics, treatment patterns, and outcomes for patients with R/R LBCL who received R‐GemOx in the second line or later (2L+) of therapy. These real‐world data can contextualize findings from global clinical trials, providing evidence for their applicability to the US population.

## 2 Methods

### 2.1 Study Design and Data Source

This retrospective, observational study used the US‐based, electronic health record (EHR)‐derived de‐identified Flatiron Health Research Database [23]. Data were derived from structured (e.g., treatment administration) and unstructured sources (e.g., clinician notes, radiology/pathology reports) available in the EHRs, with a cut‐off date for follow‐up of September 30, 2023. Initiation date of the earliest line in which the patients received R‐GemOx (index line) was defined as the index date. As the patient data included in the database were de‐identified of personal health information, based on the recommendations from the US National Institutes of Health [24], signed informed consent from patients and approval from an institutional review board were not required for this study.

### 2.2 Patient Population

A broad cohort of patients with R/R LBCL was defined from the real‐world database: Patients were eligible for inclusion if they had more than or equal to two documented clinic visits on different days, were first diagnosed with LBCL from 2011 to 2023, and received R‐GemOx in the 2L+ of therapy for R/R LBCL. The following LBCL subtypes available in the database were included: DLBCL not otherwise specified (NOS); double‐hit lymphoma; Burkitt‐like lymphoma; Epstein–Barr virus + DLBCL; gray zone lymphoma/B‐cell lymphoma, unclassifiable; primary cutaneous DLBCL (leg type); and T‐cell/histiocyte‐rich LBCL. Patients with transformations from prior indolent lymphoma were included, unless the transformation was from prior chronic lymphocytic leukemia or small lymphocytic lymphoma.

### 2.3 Study Outcomes and Analyses

The primary objective was to characterize a real‐world population of patients who received R‐GemOx for R/R LBCL in the 2L+ of therapy as routine clinical practice in the United States. Descriptive analyses included a summary of patient characteristics and treatment patterns prior to and after treatment with R‐GemOx. Overall response rate (ORR) was the primary endpoint, with secondary endpoints of complete response (CR) rate, progression‐free survival (PFS), event‐free survival (EFS), time to next treatment (TTNT), and overall survival (OS). Response assessments were based on recorded radiographic assessments and corresponding information documented in EHRs. Real‐world response was defined as a clinician assessment of a change in disease burden following radiographic imaging. Response assessment categories were captured from the clinician's qualitative description of response to therapy. Progression was assessed using the earliest evidence of disease progression from radiographic/pathologic reports, physical exam, or other clinical evidence. All efficacy outcomes were described among patients with non‐missing data for each individual endpoint.

Response outcomes were evaluated among patients with at least one documented response assessment recorded in the EHR during the index line (response‐evaluable) and with a potential for ≥ 6 months of follow‐up (clinical cut‐off date: March 30, 2023). The analyses of time‐to‐event (TTE) endpoints were assessed in all patients in the study cohort. Patients proceeding directly to consolidative ASCT or CAR T‐cell therapy were included in all TTE analyses. For PFS and OS, data analyses were performed without censoring at the time of consolidation. Initiation of consolidative therapy was treated as an event for EFS and TTNT, following standard real‐world definitions. Patients who underwent consolidative ASCT or CAR T‐cell therapy were also included in the response analyses if they had at least one response assessment during their R‐GemOx treatment line. Additional bridging treatment details can be found in the Supporting Information. Multiple sensitivity analyses were conducted to assess the robustness of the findings; full details relating to the patient populations used for analyses of response outcomes can be found in Figure S1.

TTE analyses were conducted using the Kaplan–Meier method; Sankey plots were used to visualize treatment patterns. All results are presented with 95% confidence interval (CI). The Clopper–Pearson method was used to estimate 95% CI for ORR and CR, and the Greenwood method was used to estimate 95% CI for medians of TTE endpoints. No statistical hypothesis testing was performed.

## 3 Results

### 3.1 Patient Population and Characteristics

**FIGURE 1:**
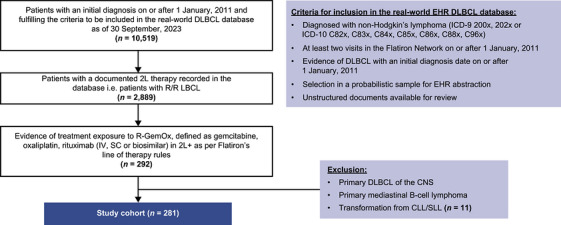
Flow chart of patients identified in the real‐world database. The following LBCL subtypes were permitted: Burkitt‐like lymphoma; DLBCL NOS; double‐hit lymphoma; EBV+ DLBCL; gray zone lymphoma/B‐cell lymphoma, unclassifiable; primary cutaneous DLBCL, (leg type); T‐cell/histiocyte‐rich LBCL. All patients, irrespective of transplant (in)eligibility, who received R‐GemOx for R/R LBCL were included. 2L, second‐line; 2L+, second or later line; CLL, chronic lymphocytic leukemia; CNS, central nervous system; DLBCL, diffuse large B‐cell lymphoma; EBV+, Epstein–Barr virus positive; EHR, electronic health records; ICD, International Classification of Diseases; IV, intravenous; LBCL, large B‐cell lymphoma; NOS, not otherwise specified; R‐GemOx, rituximab with gemcitabine and oxaliplatin; R/R, relapsed or refractory; SC, subcutaneous; SLL, small lymphocytic lymphoma.

From 2011 to 2023, 281 patients who had received R‐GemOx as 2L+ of therapy for R/R LBCL were identified in the database (Figure 1); most (66.2%) were treated at community cancer centers. The median age at R‐GemOx initiation was 71 years (range, 22–85), and 38.8% of patients were aged ≥ 75 years (Table 1). The most common LBCL diagnostic subtype reported in the database was DLBCL NOS (86.1%); 21.7% of patients had DLBCL transformed from a prior indolent malignancy, 75.4% of whom (*n* = 46/61) had transformed follicular lymphoma. Overall, 12.8% (36/281) of patients reported *MYC* and *BCL2* and/or *BCL6* rearrangements. Most patients (69.0%) had refractory disease after their first‐line treatment (primary refractory), and 76.5% were refractory to their last prior therapy. Most patients received R‐GemOx as second‐line (2L; 52.0%) or third‐line therapy (3L; 33.8%). The median number of treatment cycles was two (interquartile range, 2–5). Overall, 35% and 19% of patients completed ≥ 4 and ≥ 6 cycles, respectively.

**TABLE 1:** Patient demographics and disease characteristics at baseline.

*n* (%)	All patients,(*N* = 281)
Median age, years (range)	71 (22–85)
Age ≥ 65 years	190 (67.6)
Age ≥ 75 years	109 (38.8)
Male	176 (62.6)
Race/ethnicity^a^	(*n* = 266)
White/Black or African American/Asian	207 (77.8)/20 (7.5)/10 (3.8)
Hispanic or Latino/Not Hispanic or Latino/Other	≤ 5 (≤ 1.9)/≤ 5 (≤ 1.9)/22 (8.3)
Practice type	
Community/academic/combination of both	186 (66.2)/72 (25.6)/23 (8.2)
LBCL diagnostic subtype	
DLBCL NOS/double‐hit lymphoma/other^b^	242 (86.1)^c^/22 (7.8)/17 (6.0)
Transformation from prior indolent lymphoma	
Yes/no or unknown	61 (21.7)/220 (78.3)
Transformed from prior follicular lymphoma	46/61 (75.4)
Cell of origin	
Non‐GCB/GCB/unknown	54 (19.2)/99 (35.2)/128 (45.6)
Double‐hit status	
Yes/no or unknown	36 (12.8)/245 (87.2)
Triple‐hit status	
Yes/no or unknown	8 (2.8)/273 (97.2)
Double‐expressor status	
Yes/no or unknown	64 (22.8)/217 (77.2)
ECOG performance status	(*n = 148)*
0–1/≥ 2	105 (70.9)/43 (29.1)
Elevated lactate dehydrogenase	(*n* = 117), 77 (65.8)
Primary refractory disease^d^	194 (69.0)
Early relapse after first line of therapy^e^	34 (12.1)
Refractory to last line of therapy	215 (76.5)
Number of prior lines of therapy	
1/2/≥ 3	146 (52.0)/95 (33.8)/40 (14.2)
Median (range)	1 (1–7)
Prior therapy	
Anti‐CD20	279 (99.3)
Anthracyclines	251 (89.3)
SCT	27 (9.6)
CAR T‐cell therapy	≤ 5 (≤ 1.8)
Consolidative therapy after R‐GemOx	
Bridge to CAR T‐cell therapy	*(n = 273)* ^f^ 26 (9.5)
SCT immediately after R‐GemOx	19 (6.8)
Next anti‐lymphoma treatment	(*n = 158)* ^g^
CAR T‐cell therapy	35 (22.2)

Abbreviations: CAR, chimeric antigen receptor; DLBCL, diffuse large B‐cell lymphoma; EBV+, Epstein–Barr virus positive; ECOG, Eastern Cooperative Oncology Group; GCB, germinal center B‐cell; LBCL, large B‐cell lymphoma; NOS, not otherwise specified; R‐GemOx, rituximab with gemcitabine and oxaliplatin; SCT, stem cell transplant.

^a^ Race/ethnicity was reported as one variable; if race was missing, ethnicity was reported.

^b^ Other subtypes include: Burkitt‐like lymphoma (*n* = ≤ 5); EBV+ DLBCL (*n* = 6); gray zone lymphoma/B‐cell lymphoma, unclassifiable (*n* = 6); primary cutaneous DLBCL, leg type (*n* = ≤ 5), and T‐cell/histiocyte‐rich LBCL (*n* = ≤ 5).

^c^ Includes patients who were recorded as having DLBCL NOS but were subsequently noted to have *BCL*2/6 and *MYC* rearrangements, as determined by fluorescence in situ hybridization or karyotype testing.

^d^ Patients who did not achieve a partial or complete response to first‐line therapy and patients with documented progression within 6 months from the last known treatment during the first‐line.

^e^ Patients who achieved a partial or complete response to first‐line therapy and documented progression 6–12 months from the last known treatment during the first‐line.

^f^ Number of patients with documented evidence for use of R‐GemOx as a bridge to CAR T‐cell therapy.

^g^ Number of patients who had next line of therapy recorded in the database.

### 3.2 Treatment Patterns

Additional separate analyses (clinical cut‐off date: August 31, 2024), using the database across 3050 patients with R/R LBCL and all documented therapies in the database, found that yearly utilization of R‐GemOx as a treatment option in the 2L and 3L of therapy ranged between ≤ 3% and 9% in recent years, further highlighting its consistent use in the evolving and heterogenous treatment landscape (Figure S2). In patients with available data on duration between doses of R‐GemOx (*n* = 237), the median duration between doses of R‐GemOx (i.e., cycle length) was 2.9 weeks, with a bimodal distribution indicating the most common dosing strategies to be every 2 (Q2W; 88/237; 37.1%) or 3 (Q3W; 71/237; 30.0%) weeks (Figure S3). Baseline characteristics varied by dosing schedule; patients receiving Q3W dosing compared with Q2W were more likely to have elevated LDH (39.4% vs. 28.4%), a higher median number of prior lines of therapy (2 vs. 1), poorer performance status (ECOG PS of 2+, 22.5% vs. 15.9%), and be treated in community practices (80.3% vs. 67.0%; Table S1).

**FIGURE 2:**
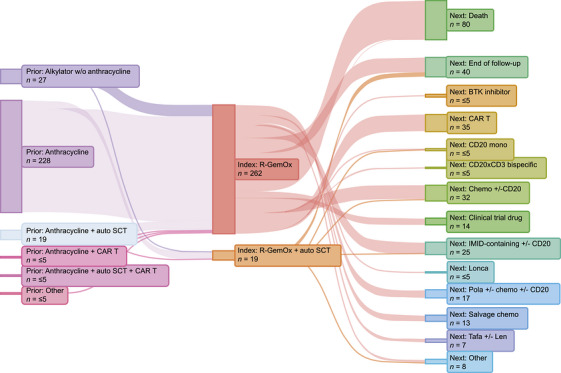
Treatments received prior to and following R‐GemOx in all patients (*N* = 281). ±, with/without; Auto SCT, autologous stem cell transplant; BTK, Bruton tyrosine kinase; CAR T, chimeric antigen receptor T‐cell therapy; chemo, chemotherapy; IMID, immunomodulatory drugs; Len, lenalidomide; Lonca, loncastuximab tesirine; Pola, polatuzumab vedotin; R‐GemOx, rituximab with gemcitabine and oxaliplatin; Tafa, tafasitamab.

Treatments received prior to R‐GemOx included anti‐CD20 therapy (99.3%), anthracyclines (89.3%), stem cell transplantation (SCT; 9.6%), and CAR T‐cell therapy (1.4%) (Figure 2; Table 1). Substantial heterogeneity was observed with regard to the next anti‐lymphoma therapy received after R‐GemOx (Figure 2). Immediate SCT was reported in 6.8% (*n* = 19/281) of patients, CAR T‐cell therapy was given in 22.2% (*n* = 35/158) of patients who had a documented next line of therapy, and the use of R‐GemOx as bridging to CAR T‐cell therapy was noted for 9.5% (*n* = 26/273) of patients (Figure 2; Table 1).

### 3.3 Treatment Outcomes With R‐GemOx

Among 185 response‐evaluable patients with a potential for ≥ 6 months of follow‐up, ORR and CR rate were 45.4% (95% CI: 38.1–52.9) and 22.2% (95% CI: 16.4–28.8), respectively (Table 2). A sensitivity analysis was conducted among all response‐evaluable patients, where deaths or lack of recorded visits within 3 months of the index date were considered as “non‐response” (*n* = 247; Figure S1). In this analysis, ORR was 34.0% (95% CI: 28.1–40.3), and the CR rate was 16.6% (95% CI: 12.2–21.8) (Table S2).

**TABLE 2:** Real‐world outcomes for R‐GemOx among patients with R/R LBCL.

Efficacy parameter	All patients, (*N* = 281)
ORR rate^a^, % (*n/N*)	45.4 (84/185)
(95% CI)	(38.1–52.9)
CR rate^a^, % (*n/N*)	22.2 (41/185)
(95% CI)	(16.4–28.8)
PFS^b^	
Median PFS, months (95% CI)	2.8 (2.6–3.7)
12‐month PFS, % (95% CI)	21.2 (16.3–27.5)
18‐month PFS, % (95% CI)	14.5 (10.4–20.4)
Median follow‐up, months (95% CI)	33.4 (18.6–61.6)
EFS^b^	
Median EFS, months (95% CI)	2.3 (2.1–2.8)
12‐month EFS, % (95% CI)	15.4 (11.5–20.4)
18‐month EFS, % (95% CI)	10.2 (7.1–14.7)
Median follow‐up, months (95% CI)	47.7 (42.4–NA)
TTNT^c^	
Median TTNT, months (95% CI)	3.9 (3.4–4.8)
12‐month TTNT, % (95% CI)	21.9 (17.4–27.7)
18‐month TTNT, % (95% CI)	14.5 (10.7–19.6)
Median follow‐up, months (95% CI)	41.6 (33.4–76.2)
OS^c^	
Median OS, months (95% CI)	12.7 (10.6–17.1)
12‐month OS, % (95% CI)	53.6 (47.6–60.2)
18‐month OS, % (95% CI)	43.0 (37.1–49.9)
Median follow‐up, months (95% CI)	37.3 (29.9–45.9)

Abbreviations: CI, confidence interval; CR, complete response; EFS, event‐free survival; LBCL, large B‐cell lymphoma; NA, not applicable; ORR, overall response rate; OS, overall survival; PFS, progression‐free survival; R/R, relapsed or refractory; R‐GemOx, rituximab with gemcitabine and oxaliplatin; TTNT, time to next treatment.

^a^ Conducted in all response‐evaluable patients with ≥ 6 months of follow‐up.

^b^ Analysis was conducted in all patients, apart from three not assessed for progression.

^c^ Conducted in all patients.

**FIGURE 3:**
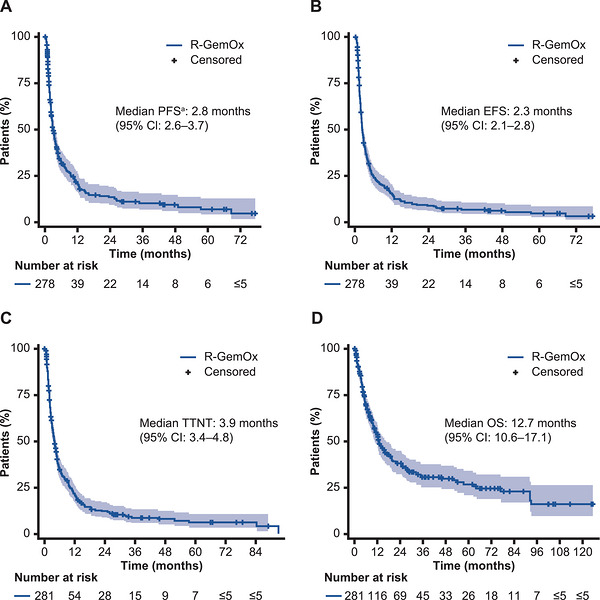
Efficacy analyses for R‐GemOx among patients with R/R LBCL: (A) PFS, (B) EFS, (C) TTNT, and (D) OS. ^a^Three patients were not assessed for progression and were not included in the PFS and EFS analyses. Shaded bands represent confidence intervals.CI, confidence interval; EFS, event‐free survival; LBCL, large B‐cell lymphoma; OS, overall survival; PFS, progression‐free survival; R/R, relapsed or refractory; R‐GemOx, rituximab with gemcitabine and oxaliplatin; TTNT, time to next treatment.

TTE outcomes were assessed in the overall cohort (*N* = 281; Table 2; Figure 3). Three patients without documented progression assessments were excluded from the PFS and EFS analyses; censoring these patients had minimal impact on outcomes (median PFS, 2.8 months with censoring vs. 3.0 months without censoring; median EFS unchanged). Median follow‐up for PFS, EFS, TTNT, and OS were 33.4, 47.7, 41.6, and 37.3 months, respectively. Median estimates (95% CI) for TTE outcomes were: 2.8 months (2.6–3.7) for PFS; 2.3 months (2.1–2.8) for EFS; 3.9 months (3.4–4.8) for TTNT, and 12.7 months (10.6–17.1) for OS. In a sensitivity analysis of TTE endpoints conducted among the 185 response‐evaluable patients with a potential for ≥ 6 months of follow‐up, TTE estimates were numerically higher than those from the main analysis, but median PFS remained low at 3.7 months in this population (Table S3). Exploratory analysis showed numerically superior survival in patients on the Q2W dosing schedule (median OS, 15.9 vs. 8.5 months; median PFS 3.4 vs. 2.7 months), though CIs overlapped (Table S4).

### 3.4 Subgroup Analyses

**FIGURE 4:**
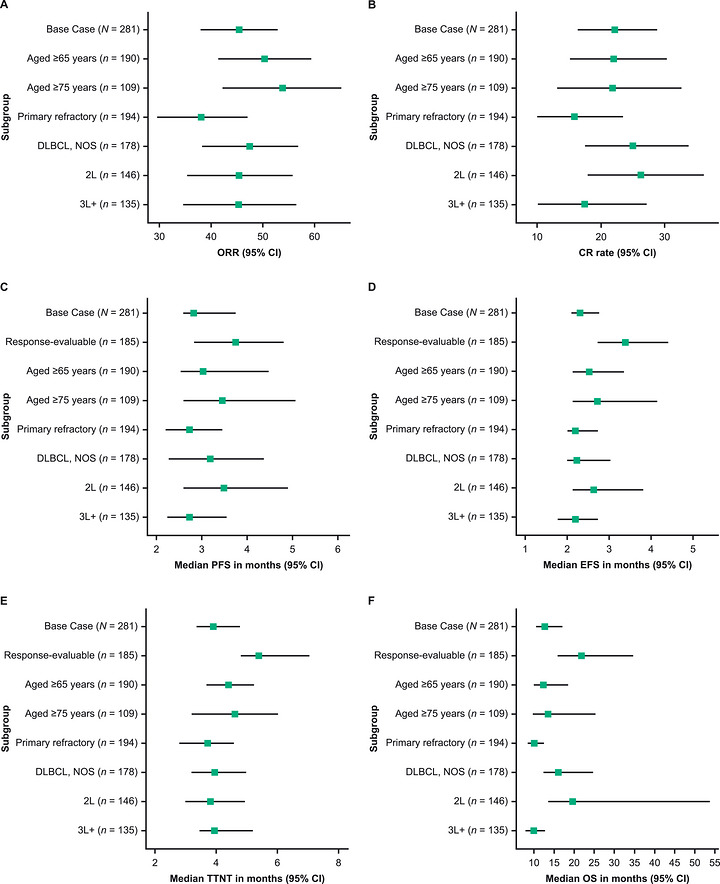
Subgroup analyses for R‐GemOx among patients with R/R LBCL: (A) ORR, (B) CR, (C) PFS, (D) EFS, (E) TTNT, and (F) OS. Green solid squares indicate point estimates, black horizontal lines indicate 95% CI for the point estimate. Main analyses for ORR and CR were conducted in response‐evaluable patients with ≥ 6 months of follow‐up. Subgroup analyses of ORR and CR for patients with primary refractory disease, R‐GemOx by line of therapy, elderly patients, and patients with DLBCL NOS subtype were conducted in all response‐evaluable patients with any length of follow‐up. These analyses are exploratory. Main analysis for PFS, EFS, TTNT, and OS was conducted in all patients in the study cohort. Three patients were not assessed for progression and were not included in the PFS and EFS analyses. 2L, second‐line; 3L+, third or later line; CI, confidence interval; CR, complete response; DLBCL, diffuse large B‐cell lymphoma; EFS, event‐free survival; LBCL, large B‐cell lymphoma; NOS, not otherwise specified; ORR, overall response rate; OS, overall survival; PFS, progression‐free survival; R‐GemOx, rituximab with gemcitabine and oxaliplatin; R/R, relapsed or refractory; TTNT, time to next treatment.

Subgroup analyses were performed (Figure 4) among patients with advanced age (aged ≥ 65 and ≥ 75 years), primary refractory disease, DLBCL NOS subtype, and receiving R‐GemOx in the 2L and 3L+ (third or later line). Patient characteristics within the subgroups and outcomes are described in Tables S5 and S6. The majority (64.4%) of patients with primary refractory disease to the first‐line treatment were aged ≥ 65 years and almost all (95.9%) were also refractory to their last prior therapy. Compared with the broader study cohort, less patients with DLBCL NOS disease were primary refractory or refractory to their last prior therapy than patients in other subgroups. More patients with poorer prognostic characteristics, such as primary refractory disease or refractoriness to last prior therapy received R‐GemOx as a 3L+ therapy. Across all subgroup analyses, observed differences among baseline characteristics suggested mostly numerical differences in outcomes. However, patients with primary refractory disease had poorer ORR, CR rate, and OS compared with the broader study cohort (Figure 4). In addition, patients receiving R‐GemOx in the 2L experienced longer OS than patients receiving R‐GemOx in the 3L+. Among patients not receiving R‐GemOx for bridging therapy, the CR rate and median OS were numerically lower than the overall cohort (17.9% vs. 22.2% and 10.6 vs. 12.7, respectively), while the other responses were comparable (Table S6).

## 4 Discussion

Using real‐world data from a large US‐based, EHR‐derived database, this study reports the characteristics, treatment patterns, and outcomes of 281 patients with R/R LBCL who received R‐GemOx as 2L+ therapy between 2011 and 2023. This represents the largest cohort of patients to date who have been treated with R‐GemOx in the United States, predominantly in community cancer centers.

From 2011 to 2024, the database showed consistent use of R‐GemOx as 2L+ therapy, supporting its relevance as a standard of care regimen despite the evolving R/R LBCL treatment landscape and availability of newer therapy options. More than half (52%) of the patients in this study received R‐GemOx as a 2L therapy. As expected, the most common therapies received prior to R‐GemOx were anti‐CD20 therapies (99%) and anthracycline‐containing regimens (89%), which are the mainstay of therapy for LBCL [1]. Only a small proportion of patients received SCT or CAR T‐cell therapy prior to R‐GemOx, highlighting the importance of alternative treatments for patients who are ineligible for ASCT or CAR T‐cell therapy [15].

There was considerable heterogeneity observed in the next anti‐lymphoma therapy received after R‐GemOx; a small group of patients received SCT immediately following R‐GemOx, and some received R‐GemOx as bridging to CAR T‐cell therapy. Overall, CAR T‐cell therapy was the most common next line of therapy, but was only given in 22% of patients. This indicates a lack of defined treatment pathways and an unmet medical need for patients with R/R LBCL.

Real‐world outcomes of effectiveness reported in the current study characterize the use of R‐GemOx as a commonly used treatment for patients with R/R LBCL, demonstrating an ORR of 45%, with 22% of patients having a CR. Median PFS, EFS, TTNT, and OS were 2.8, 2.3, 3.9, and 12.7 months, respectively. It is important to note that the assessment of ORR and CR in this study was limited to patients with at least one recorded response assessment in EHRs, while TTE outcomes were assessed in all patients. Approximately 32% of the total cohort had no documented response assessment. This reflects the reality of community‐based EHR data where scans from external facilities may not be captured. Therefore, estimates of ORR and CR are subject to guarantee‐time bias by excluding patients who had early progressions without a formal response assessment. We believe that the true real‐world ORR likely falls between the 34.0% and 45.4% estimates.

When contextualizing these results with previously published cohorts, denominator selection substantially impacts the perceived effectiveness of R‐GemOx. The ORR was 45.4% in the response‐evaluable population (*n* = 185), but the more conservative all‐treated sensitivity analysis (*n* = 247) yielded an ORR of 34.0%, which may provide a more appropriate comparison with prior studies. The LEO‐CReWE study recently reported outcomes from a cohort of patients with R/R DLBCL (*N* = 183) from eight academic centers in the United States, between 2007 and 2022: [25] ORR was 45%, CR rate was 29%, median EFS was 2.3 months, and median OS was 13.5 months. These results suggest that although response outcomes were lower in our predominantly community‐based cohort, patient TTE outcomes with R‐GemOx are largely consistent across different care settings in the United States. In a retrospective study of transplant‐ineligible patients with R/R DLBCL treated with R‐GemOx in France between 2002 and 2017 (*N* = 196), the ORR and CR rates were 54% and 23%, and median PFS and OS were 5 and 10 months, respectively [26].

Two US‐based real‐world studies reported poorer outcomes with R‐GemOx than the current study [27, 28]. Garg et al. conducted a retrospective analysis of Medicare claims in the United States from 2014 to 2019 and identified 157 patients > 65 years old who received treatment with R‐GemOx in the R/R DLBCL setting [27]. ORR and CR rates were not reported but median OS was 6.9 months in the 2L setting and 6.8 months in the 3L setting. Schade et al. conducted a single‐center real‐world study from 2007 to 2018 and reported outcomes for patients with any R/R aggressive B‐cell non‐Hodgkin lymphoma who were treated with GemOx, with or without rituximab [28]. Of 140 patients, 81 received R‐GemOx and 59 received GemOx alone; ORR was 26%, CR rate was 15%, and median PFS and OS were 1.4 and 7.8 months, respectively. The poorer outcomes reported in these studies, compared with the current study, may be due to the earlier timeframes assessed in the former, representative of an older treatment landscape, as well as potential differences in data sources and patient populations chosen.

While outcomes reported in the current study appear to align with those of R‐GemOx control arms from recent prospective phase III clinical trials, these comparisons must be interpreted with caution due to fundamental methodological differences in clinical assessment and patient selection. In the R‐GemOx arm of the STARGLO trial, ORR was 41%, CR rate was 25%, median PFS was 3.6 months, and median OS was 12.9 months [17, 29]. In the R‐GemOx arm of the NIVEAU trial, ORR and CR rate were 34% and 20%, respectively; the 1‐year PFS rate was 28% and 1‐year OS rate was 48% [22]. The POLARGO trial reported an ORR of 31% and CR rate of 24% at the end of treatment and a median PFS and OS of 2.7 and 12.5 months, respectively for R‐GemOx [30]. Finally, the SUNMO trial reported an ORR of 40% and CR rate of 24% with median PFS of 3.8 months for R‐GemOx [31].

The current study assessed outcomes by dosing schedule. Exploratory analyses suggested numerically longer OS and PFS among patients receiving Q2W versus Q3W dosing. However, CIs overlapped, and the differences in baseline patient fitness and treatment intent may have influenced outcomes between groups. Outcomes were also assessed in specific subgroups of patients. Median OS was approximately twice as long in patients who received R‐GemOx in the 2L, compared with those who received it in the 3L+ (19.6 vs. 10.0 months, respectively). Similarly, CR rates were higher for patients who received R‐GemOx in the 2L versus the 3L+ (26% vs. 17%, respectively). These findings may be explained due to the worsening prognosis of patients with subsequent lines of therapy, and the limited availability of effective treatment options, supporting the development of novel regimens for this patient population. The CR rate and median OS in the non‐bridging subgroup suggests that a portion of the long‐term clinical benefit observed in the total cohort is attributable to subsequent intensive therapies rather than R‐GemOx alone. However, the consistent ORR and median PFS indicates that the initial disease control provided by R‐GemOx is consistent regardless of bridging intent.

This study had several limitations. First, real‐world data extracted from EHRs are not intended for research purposes and are not as robust as clinical trial data. Specifically, response and progression data were assessed neither using prespecified and standardized methodology, such as the Lugano 2014 criteria [32], nor assessed by centralized/independent review. Due to inconsistencies and irregular clinic visits in routine clinical practice, there was a high level of missing data in the real‐world database, including a disproportionally high level of missing data for response outcomes compared with TTE endpoints [33]. Consequently, different analysis populations were used in this study to assess efficacy outcomes. As such, multiple sensitivity analyses were conducted to assess the robustness of the results. A sensitivity analysis conducted among all response‐evaluable patients and attributing deaths or lack of recorded visits within 3 months of the index date as “non‐response” indicated that there were a considerable number of patients who died or were lost to follow‐up prior to being assessed for response. However, it is reasonable to assume that the true real‐world response rates will be within the range of estimates calculated in the main analysis and in the sensitivity analyses. Information on transplant (in)eligibility was not readily available from the real‐world database, and, therefore, analyses focusing solely on transplant‐ineligible patients could not be conducted. Data on relative dose intensity and specific reasons for discontinuation (e.g., toxicity vs. progression) were not captured, which limits our ability to fully characterize treatment intensity in this cohort. Finally, the current study was not designed to assess the safety of R‐GemOx, which is an important real‐world consideration for clinicians and patients. Future studies that include safety endpoints, such as treatment‐related adverse events, will be of significant interest.

In conclusion, to date, this is the largest cohort of patients that characterizes the real‐world use of R‐GemOx in patients with R/R LBCL in the United States who have primarily received care in community cancer centers. These data demonstrate that R‐GemOx has been used as a standard‐of‐care treatment option in the heterogenous and continuously evolving R/R LBCL setting and provides a real‐world perspective on its outcomes in the United States. Furthermore, the data from the present study help to contextualize the findings from global trials using R‐GemOx as a comparator arm and their applicability to patients with R/R LBCL in the United States.

## Author Contributions

M.C.W., L.L, A.S., N.S., N.P., S.Y., and C.L.B. contributed to the conception and design of the study. A.S., N.S., and N.P. provided study materials or patients. A.S., N.S., and N.P. collected and assembled the data. All authors analyzed and interpreted the data, contributed to the writing of and provided final approval of the manuscript, and were accountable for all aspects of the work.

## Funding

This real‐world data study is sponsored by Genentech, Inc./F. Hoffmann‐La Roche Ltd.

## Ethics Statement

This research uses secondary data from the Flatiron Health Research Database, which was de‐identified and therefore not considered human subjects research. Based on recommendations from the National Institute of Health, this research did not require review/approval from an institutional review board.

## Consent

As patient data were de‐identified of personal health information, signed informed consent from patients was not required for this study.

## Conflicts of Interest

Elizabeth Budde: grants or contracts: AstraZeneca, Merck, consulting fees: Genentech, Inc./F. Hoffmann‐La Roche Ltd, Kite Pharma, AstraZeneca, Regeneron, ADC Therapeutics, AbbVie, Jessen, Foresight, Bristol Myers Squibb, other: current employment at City of Hope; Adam J. Olszewski: grants or contracts: Genentech, Inc./F. Hoffmann‐La Roche Ltd, Genmab, Artiva, Pfizer, Kymera Therapeutics, NIGMS grant P30GM145500, consulting fees: Genentech, Inc./F. Hoffmann‐La Roche Ltd, Schrodinger, participation in a data safety monitoring board or advisory board: Genmab, ADC Therapeutics, BeiGene, Bristol Myers Squibb. Bei Hu: grants or contracts: Genentech, Inc., BeiGene, Bristol Myers Squibb, consulting fees: Janssen Biotech. Nikesh N. Shah: speaker bureau: Amgen, Takeda, Jazz, Genentech, Inc., Servier, advisory board: Bristol Myers Squibb, ADC Therapeutics. Connie Lee Batlevi: stock or stock options: F. Hoffmann‐La Roche Ltd, other financial or nonfinancial interests: employee of Genentech, Inc./F. Hoffmann‐La Roche Ltd. Shen Yin: support for attending meetings and/or travel: Genentech, Inc./F. Hoffmann‐La Roche Ltd, patents planned, issued, or pending: Genentech, Inc./F. Hoffmann‐La Roche Ltd; stock or stock options: F. Hoffmann‐La Roche Ltd, other financial or nonfinancial interests: Genentech, Inc./F. Hoffmann‐La Roche Ltd. Iris To: support for attending meetings and/or travel: Genentech, Inc./F. Hoffmann‐La Roche Ltd, patents planned, issued, or pending: Genentech, Inc./F. Hoffmann‐La Roche Ltd, stocks or stock options: Genentech, Inc./F. Hoffmann‐La Roche Ltd. Michael C. Wei: support for attending meetings and/or travel: Genentech, Inc., patents planned, issued, or pending: Genentech, Inc., stock or stock options: F. Hoffmann‐La Roche Ltd, other: Genentech, Inc. Martine Joanna Kallemeijn: stock or stock options: Roche Connect equities. Lisa Musick: other: employee of F. Hoffmann‐La Roche Ltd. Linda Lundberg: patents planned, issued or pending: F. Hoffmann‐La Roche Ltd; stock or stock options: F. Hoffmann‐La Roche Ltd, other: employee of F. Hoffmann‐La Roche Ltd. Natasha Shamas: stock or stock options: Genentech, Inc./F. Hoffmann‐La Roche Ltd. Mark Dixon: patents planned, issued or pending: F. Hoffmann‐La Roche Ltd, stocks or stock options: F. Hoffmann‐La Roche Ltd, other: employee of Roche Products Limited. Navdeep Pal: stock or stock options: Genentech, Inc./F. Hoffmann‐La Roche Ltd, other: Genentech, Inc./F. Hoffmann‐La Roche Ltd. Ashwini Shewade: support for attending meetings and/or travel: Genentech, Inc., stock or stock options: F. Hoffmann‐La Roche Ltd, other: employee of Genentech, Inc., a member of Roche Group. Jason Westin: grants or contracts: AstraZeneca, Nurix, Janssen, Morphosys/Incyte, ADC Therapeutics, Bristol Myers Squibb, Genentech, Inc., Kite/Gilead, Allogene, Novartis, Regeneron, consulting fees: AbbVie/GenMab, AstraZeneca, Nurix, Janssen, Morphosys/Incyte, ADC Therapeutics, Bristol Myers Squibb, Genentech, Inc., Kite/Gilead, Allogene, Novartis, Regeneron, Pfizer, Lyell.

## Supporting information




**Supporting file 1**: jha270351‐sup‐0001‐Appendix.docx

## Data Availability

The data that support the findings of this study have been originated by Flatiron Health, Inc. These de‐identified data may be made available upon request, and are subject to a license agreement with Flatiron Health; interested researchers should contact DataAcces@flatiron.com to determine licensing terms.
