# Supplementary material for: A Real‐World Study Characterizing a US Population of Patients Receiving Rituximab With Gemcitabine and Oxaliplatin for Relapsed or Refractory Large B‐Cell Lymphoma
Source: EJHaem. 2026 Aug 13;7(4):e70351. doi: 10.1002/jha2.70351 (PMC13472062; doi:10.1002/jha2.70351)
Supplement: Supplementary file 1 [file JHA2-7-e70351-s001.docx]

**APPENDIX**

**Time to event endpoint definitions**

Progression-free survival was defined as the time from initiation of the index therapy until first documented progression or death. Real-world progression was defined as a distinct episode in the patient journey at which the treating clinician concluded there was spread or worsening in the underlying disease based on radiographic and/or pathologic and/or physical exam and/or other non-specific clinical assessment. In absence of documented progression during available follow-up for the index line of therapy, the patient was censored at the earliest start of the next anti-lymphoma treatment or last clinic note date recorded in the electronic health records (EHR) for assessment of progression.

Event-free survival was defined as the time from initiation of the index therapy until the earliest of first documented progression or initiation of next/subsequent anti-lymphoma therapy or death. If none of those events were documented in the EHR, the patient was censored at the earliest of start of next anti-lymphoma treatment or last clinic note date recorded in the EHR for assessment of progression.

Time to next anti-lymphoma treatment was defined as time from the initiation of index therapy until the initiation of the next or subsequent anti-lymphoma therapy or death. If no subsequent line of therapy was documented, the patient was censored at the documented last visit date in the EHR.

**Sensitivity analyses for efficacy outcomes**

Sensitivity analyses were conducted to determine accurate real-world outcomes and the robustness of results. Figure S1 summarises the distribution of patients that were considered for the main case and three sensitivity analyses of response outcomes. Overall and complete response rates are reported in Table S2. Sensitivity analyses 1 and 2 were comparable to the main analysis. However, the results of sensitivity analysis 3 were lower than the main analysis.

Sensitivity analyses of time-to-event outcomes are reported in Table S3. Compared with the main analysis, outcomes were improved with sensitivity analysis 4 and remained consistent with sensitivity analysis 5.

**Rituximab with gemcitabine and oxaliplatin (R-GemOx) as bridging therapy**

Patients were classified as receiving bridging therapy if they met at least one of the following criteria: (1) a documented clinician flag in the EHR indicating bridging intent; (2) receipt of autologous or allogeneic stem cell transplant during the index R-GemOx line of therapy; or (3) initiation of chimeric antigen receptor (CAR) T-cell therapy as the immediate next line of therapy following R-GemOx. This multi-modal definition was used to account for both documented clinical intent and observed treatment patterns. These patients were excluded from a subgroup analysis of response and time-to-event endpoints.

**TABLE S1. Baseline characteristics of R-GemOx patients on Q2W or Q3W cycle frequencies**

| ***n* (%)** | **R-GemOx Q2W  (*n* = 88)** | **R-GemOx Q3W  (*n* = 71)** |
| --- | --- | --- |
| **Median age,** years (range) | 72 (22–85) | 72 (44–85) |
| Age ≥65 years | 64 (72.7) | 51 (71.8) |
| **Male** | 51 (58.0) | 46 (64.8) |
| **Race/ethnicity** |  |  |
| White | 67 (76.1) | 49 (69.0) |
| Black or African American | 7 (8.0) | 3 (4.2) |
| Asian | 3 (3.4) | 1 (1.4) |
| Other | 8 (9.2) | 9 (12.7) |
| **Practice type** |  |  |
| Community | 59 (67.0) | 57 (80.3) |
| Academic | 16 (18.2) | 11 (15.5) |
| Combination of both | 13 (14.8) | 3 (4.2) |
| **LBCL diagnostic subtype** |  |  |
| DLBCL NOS | 76 (86.4) | 63 (88.7) |
| Double-hit lymphoma | 10 (11.4) | 8 (11.3) |
| **Transformation from prior indolent lymphoma** |  |  |
| Yes | 15 (17.0) | 18 (25.4) |
| No or unknown | 73 (83.0) | 53 (74.6) |
| Transformed from prior follicular lymphoma | 11 (12,5) | 16 (22.5) |
| **Transplant in index line** | 9 (10.2) | 1 (1.4) |
| **Cell of origin** |  |  |
| Non-GCB/ABC | 15 (17.0) | 18 (25.4) |
| GCB | 31 (35.2) | 23 (32.4) |
| Unknown | 42 (47.7) | 30 (42.3) |
| **Double-hit status** |  |  |
| Yes | 10 (11.4) | 8 (11.3) |
| No or unknown | 78 (88.6) | 63 (88.7) |
| **Triple-hit status** |  |  |
| Yes | 1 (1.1) | 1 (1.4) |
| No | 50 (56.8) | 31 (43.7) |
| Unknown | 37 (42.0) | 39 (54.9) |
| **Double-expressor status** |  |  |
| Yes | 19 (21.6) | 15 (21.1) |
| No or unknown | 69 (78.4) | 56 (79.9) |
| **ECOG performance status** |  |  |
| 0 | 19 (21.6) | 13 (18.3) |
| 1 | 28 (31.8) | 19 (26.8) |
| ≥2 | 14 (15.9) | 16 (22.5) |
| **Elevated lactate dehydrogenase** | 25 (28.4) | 28 (39.4) |
| **Primary refractory disease** | 58 (65.9) | 54 (76.1) |
| **Early relapse after first line  of therapy** | 10 (11.4) | 7 9.9) |
| **Refractory to last line of therapy** | 65 (73.9) | 58 (81.7) |
| **Number of prior lines of therapy** |  |  |
| 1 | 51 (58.0) | 33 (46.5) |
| 2 | 24 (27.3) | 28 (39.4) |
| ≥3 | 13 (14.8) | 10 (14.1) |
| Median (range) | 1 (1–4) | 2 (1–7) |
| **Prior therapy** |  |  |
| Anti-CD20 | 87 (98.9) | 70 (98.6) |
| Anthracyclines | 79 (89.8) | 62 (87.3) |
| SCT | 5 (5.7) | 4 (5.6) |
| CAR T-cell therapy | 1 (1.1) | 0 |
| **Evidence of bridging therapy** | 7 (8.0) | 5 (7.0) |
| **Median number of cycles, (range)** | 4.5 (2–13) | 4 (2–13) |
| Completed ≥4 cycles | 53 (60.2) | 37 (52.1) |
| Completed ≥6 cycles | 31 (35.2) | 20 (28.2) |

Abbreviations: DLBCL, diffuse large B-cell lymphoma; ECOG, Eastern Cooperative Oncology Group; LDH, lactate dehydrogenase; NOS, not otherwise specified; Q2W, every 2 weeks; Q3W, every 3 weeks; R-GemOx, rituximab, gemcitabine, and oxaliplatin; SCT, stem cell transplant.

**TABLE S2.** **Sensitivity analyses for R-GemOx ORR and CR rates among patients with R/R LBCL.**

| **Patient population assessed in response  outcome analyses** | **ORR,** % (*n/N*)  [95% CI] | **CR,** % (*n/N*)  [95% CI] |
| --- | --- | --- |
| **Main analysis**  *Response-evaluable patients with ≥6 months  of follow-up^a^* | 45.4 (84/185)  [38.1–52.9] | 22.2 (41/185)  [16.4–28.8] |
| **Sensitivity analysis 1** *Response-evaluable patients with ≥3 months  of follow-up^a^* | 44.4 (84/189)  [37.2–51.8] | 21.7 (41/189)  [16.0–28.3] |
| **Sensitivity analysis 2** *Response-evaluable patients with* *any length  of follow-up^a^* | 44.0 (84/191)  [36.8–51.3] | 21.5 (41/191)  [15.9–28.0] |
| **Sensitivity analysis 3**  *Response-evaluable patients^a^ and attributing deaths or lack of recorded visits within 3 months of the index date as “non-response”^b^* | 34.0 (84/247)  [28.1–40.3] | 16.6 (41/247)  [12.2–21.8] |

Abbreviations: CR, complete response; EHR, electronic health records, LBCL, large B-cell lymphoma; ORR, overall response rate; R-GemOx, rituximab with gemcitabine and oxaliplatin; R/R, relapsed or refractory.

^a^Patients with at least one documented tumour assessment recorded in the EHR during the index line.

^b^Overall, 34 patients either did not have any recorded response assessment during follow-up but were not lost to follow-up or did not die within 3 months of the index date.

**TABLE S3.** **Sensitivity analyses for R-GemOx TTE outcomes among patients with R/R LBCL.**

| **TTE outcome, months (95% CI)** | **Main analysis**  All patients  **(*N* = 281)** | **Sensitivity analysis 4**  Response-evaluable patients with  ≥6 months of follow-up^a^  **(*n* = 185)** | **Sensitivity analysis 5**  Additional censoring at the start of a visit gap of ≥3 months during follow-up **(*N* = 281)** |
| --- | --- | --- | --- |
| **Median PFS**^b^ | 2.8 (2.6–3.7) | 3.7 (2.8–4.8) | 2.8 (2.6–3.7) |
| **Median EFS**^b^ | 2.3 (2.1–2.8) | 3.4 (2.7–4.4) | 2.3 (2.1–3.8) |
| **Median TTNT** | 3.9 (3.4–4.8) | 5.4 (4.8–7.0) | 3.9 (3.4–4.8) |
| **Median OS** | 12.7 (10.6–17.1) | 21.9 (15.9–34.7) | NA |

Abbreviations: CR, complete response; EFS, event-free survival; LBCL, large B-cell lymphoma; NA, not applicable; ORR, overall response rate; OS, overall survival; PFS, progression-free survival; R-GemOx, rituximab with gemcitabine and oxaliplatin; R/R, relapsed or refractory; TTE, time-to-event; TTNT, time to next treatment.

^a^Same patient population used for main analysis of ORR and CR rate.

^b^Three patients were not assessed for progression and not included in the analyses.

**TABLE S4. Exploratory efficacy outcomes for R-GemOx patients on Q2W or Q3W cycle frequencies.**

| **TTE outcome, months (95% CI)** | **R-GemOx**  **Q2W**  **(*n* = 88)** | **R-GemOx Q3W  (*n* = 71)** |
| --- | --- | --- |
| **Median PFS** | 3.4 (2.2–7.1) | 2.7 (2.1–3.8) |
| **Median EFS** | 2.3 (1.9–3.7) | 2.3 (1.9–3.5) |
| **Median TTNALT** | 5.3 (3.0–9.8) | 3.8 (3.0–4.9) |
| **Median OS** | 15.9 (10.1–34.7) | 8.5 (5.4–12.5) |
| **ORR,** % (n/N) [95% CI] | 57.4 (35/61) [44.1–70.0] | 34.5 (19/55) [22.2–48.6] |
| **CR rate,** % (n/N) [95% CI] | 26.2 (16/61) [15.8–39.1] | 12.7 (7/55) [5.3–24.5] |

Response rates among response-evaluable patients with at least 6 months of potential follow-up.

Abbreviations: CI, confidence interval; CR, complete response; EFS, event-free survival; ORR, overall response rate; OS, overall survival; PFS, progression-free survival; Q2W, every 2 weeks; Q3W, every 3 weeks; R-GemOx, rituximab, gemcitabine, and oxaliplatin; TTE, time-to-event; TTNALT, time to next anti-lymphoma treatment.

**TABLE S5.** **Patient demographics and disease characteristics at baseline, in subgroups of interest.**

| ***n* (%)** | **R-GemOx in the 2L  (*n* = 146)** | **R-GemOx in the 3L+  (*n* = 135)** | **Aged ≥65 years**  **(*n* = 190)** | **Aged ≥75 years**  **(*n* = 109)** | **Primary refractory disease**  **(*n* = 194)** | **DLBCL NOS    (*n* = 178)^a^** |
| --- | --- | --- | --- | --- | --- | --- |
| **Median age,**^b^ years (range) | 74 (39–85) | 67 (22–85) | 75 (65–85) | 79 (75–85) | 69 (22–85) | 71 (28–85) |
| Age ≥65 years | 109 (74.7) | 81 (60.0) | 190 (100) | 109 (100) | 125 (64.4) | 126 (70.8) |
| **Male** | 88 (60.3) | 88 (65.2) | 110 (57.9) | 58 (53.2) | 129 (66.5) | 106 (59.6) |
| **Race/ethnicity**^c^ | *(n = 135)* | *(n = 131)* | *(n = 180)* | *(n = 100)* | *(n = 183)* | *(n = 167)* |
| White | 108 (80.0) | 99 (75.6) | 147 (81.7) | 82 (82.0) | 140 (76.5) | 128 (76.6) |
| Black or African American | 9 (6.7) | 11 (8.4) | 10 (5.6) | ≤5 (≤5.0) | 15 (8.2) | 13 (7.8) |
| Asian | ≤5 (≤3.7) | 8 (6.1) | ≤5 (≤2.8) | ≤5 (≤5.0) | 6 (3.3) | 6 (3.4) |
| Hispanic or Latino | 0 | ≤5 (≤3.8) | 0 | 0 | 6 (3.3) | 0 |
| Not Hispanic or Latino | ≤5 (≤3.7) | ≤5 (≤3.8) | ≤5 (≤2.8) | ≤5 (≤5.0) | ≤5 (≤2.7) | ≤5 (≤3.0) |
| Other | 13 (9.6) | 9 (6.9) | 16 (8.9) | 10 (10.0) | 15 (8.2) | 16 (9.6) |
| **Practice type** |  |  |  |  |  |  |
| Community | 100 (68.5) | 86 (63.7) | 126 (66.3) | 71 (65.1) | 134 (69.1) | 117 (65.7) |
| Academic | 36 (24.7) | 36 (26.7) | 46 (24.2) | 27 (24.8) | 44 (22.7) | 45 (25.3) |
| Combination of both | 10 (6.8) | 13 (9.6) | 18 (9.5) | 11 (10.1) | 16 (8.2) | 16 (9.0) |
| **LBCL diagnostic subtype** |  |  |  |  |  |  |
| DLBCL NOS | 128 (87.7) | 114 (84.4) | 169 (89.0) | 98 (89.9) | 163 (84.0) | 178 (100) |
| Double-hit lymphoma | 7 (4.8) | 15 (11.1) | 12 (6.3) | 8 (7.4) | 19 (9.8) | 0 |
| Other^d^ | 11 (7.5) | 6 (4.4) | 9 (4.7) | ≤5 (≤4.6) | 12 (6.2) | 0 |
| **Transformation from prior indolent lymphoma** |  |  |  |  |  |  |
| Yes | 34 (23.3) | 27 (20.0) | 39 (20.5) | 22 (20.2) | 44 (22.7) | 0 |
| No or unknown | 112 (76.7) | 108 (80.0) | 151 (79.5) | 87 (79.8) | 150 (77.3) | 178 (100) |
| Transformed from prior follicular lymphoma | 25/34 (73.5) | 21/27 (77.8) | 29/39 (74.4) | 15/22 (68.2) | 150 (77.3) | 0 |
| **Cell of origin** |  |  |  |  |  |  |
| Non-GCB | 26 (17.8) | 28 (20.7) | 36 (18.9) | 20 (18.3) | 34 (17.5) | 44 (24.7) |
| GCB | 56 (38.4) | 43 (31.9) | 69 (36.3) | 38 (34.9) | 73 (37.6) | 66 (37.1) |
| Unknown | 64 (43.8) | 64 (47.4) | 85 (44.7) | 51 (46.8) | 87 (44.8) | 68 (38.2) |
| **Double-hit status** |  |  |  |  |  |  |
| Yes | 13 (8.9) | 23 (17.0) | 23 (12.1) | 15 (13.8) | 29 (14.9) | 0 |
| No or unknown | 133 (91.1) | 112 (83.0) | 167 (87.9) | 94 (86.2) | 165 (85.1) | 178 (100) |
| **Triple-hit status** |  |  |  |  |  |  |
| Yes | ≤5 (≤3.4) | ≤5 (≤3.7) / 131 | 7 (2.7) | ≤5 (≤4.6) | 165 (85.1) | 0 |
| No or unknown | 142 (97.3) | (97.0) | 183 (96.3) | 104 (95.4) | 189 (97.4) | 178 (100) |
| **Double-expressor status** |  |  |  |  |  |  |
| Yes | 32 (21.9) | 32 (23.7) | 45 (23.7) | 26 (23.9) | 44 (22.7) | 37 (20.8) |
| No or unknown | 114 (78.1) | 103 (76.3) | 145 (76.3) | 83 (76.1) | 150 (77.3) | 141 (79.2) |
| **ECOG performance status** | *(n = 78)* | *(n = 70)* | *(n = 111)* | *(n = 65)* | *(n = 103)* | *(n = 87)* |
| 0–1 | 59 (75.6) | 46 (65.7) | 77 (69.4) | 46 (70.8) | 73 (70.9) | 60 (69.0) |
| ≥2 | 19 (24.4) | 24 (34.3) | 34 (30.6) | 19 (29.2) | 30 (29.1) | 27 (31.0) |
| **Elevated lactate dehydrogenase** | *(n = 61)* 39 (63.9) | *(n = 56)* 38 (67.9) | *(n = 86)*  56 (65.1) | *(n = 48)*  32 (66.7) | *(n = 82)*  61 (74.4) | *(n = 76)*  47 (61.8) |
| **Primary refractory disease**^e^ | 96 (65.8) | 98 (72.6) | 125 (65.8) | 67 (61.5) | 194 (100) | 117 (65.7) |
| **Early relapse after first line  of therapy**^f^ | 17 (11.6) | 17 (12.6) | 25 (13.2) | 15 (13.8) | 0 | 21 (11.8) |
| **Refractory to last line of therapy** | 96 (65.8) | 119 (88.1) | 139 (73.2) | 76 (69.7) | 186 (95.9) | 131 (73.6) |
| **Number of prior lines of therapy** |  |  |  |  |  |  |
| 1 | 146 (100) | 0 | 109 (57.4) | 69 (63.3) | 96 (49.5) | 94 (52.8) |
| 2 | 0 | 95 (70.4) | 61 (32.1) | 32 (29.4) | 65 (33.5) | 61 (34.3) |
| ≥3 | 0 | 40 (29.6) | 20 (10.5) | 8 (7.3) | 33 (17.0) | 23 (12.9) |
| Median (range) | 1 (1–1) | 2 (2–7) | 1 (1–7) | 1 (1–3) | 2 (1–7) | 1 (1–7) |
| **Prior therapy** |  |  |  |  |  |  |
| Anti-CD20 | 144 (98.6) | 135 (100) | 190 (100) | 109 (100) | 192 (99.0) | 177 (99.4) |
| Anthracyclines | 129 (88.4) | 122 (90.4) | 170 (89.5) | 98 (89.9) | 170 (87.6) | 165 (92.7) |
| SCT | ≤5 (≤3.4) | 22 (16.3) | 12 (6.3) | ≤5 (≤4.6) | 18 (9.3) | 20 (11.2) |
| CAR T-cell therapy | 0 | ≤5 (≤3.7) | ≤5 (≤2.6) | ≤5 (≤4.6) | ≤5 (≤2.6) | ≤5 (≤2.8) |
| **SCT immediately after R-GemOx** | 14 (9.6) | ≤5 (≤3.7) | 10 (5.3) | ≤5 (≤4.6) | 9 (4.6) | 10 (5.6) |
| **Next anti-lymphoma treatment**^g^ | *(n = 84)* | *(n = 74)* | *(n = 99)* | *(n = 60)* | *(n = 106)* | *(n = 105)* |
| CAR T-cell therapy | 21 (25.0) | 14 (18.9) | 23 (23.2) | 11 (18.3%) | 23 (21.7) | 25 (23.8) |
| **Bridge to CAR T-cell therapy**^h^ | *(n = 142)*  13 (9.2) | *(n = 131)* 13 (9.9) | *(n = 185)*  16 (8.6) | *(n = 108)*  ≤5 (≤4.6) | *(n = 188)*  19 (10.1) | *(n = 173)*  19 (11.0) |

Abbreviations: 2L, second line; 3L+, third line or later; CAR, chimeric antigen receptor; DLBCL, diffuse large B-cell lymphoma; EBV+, Epstein-Barr virus positive; ECOG, Eastern Cooperative Oncology Group; GCB, germinal center B-cell; LBCL, large B-cell lymphoma; NOS, not otherwise specified; R-GemOx, rituximab with gemcitabine and oxaliplatin; SCT, stem cell transplant.

^a^For the DLBCL NOS disease subgroup analysis, patients who had *BCL*2/6 and *MYC* rearrangements were removed from the DLBCL NOS subgroup to get a “true” DLBCL NOS representation.

^b^Values above 85 are suppressed at 85 to preserve patient deidentification.

^c^Race/ethnicity was reported as one variable; if race was missing, ethnicity was reported.
^d^Other subtypes include: Burkitt-like lymphoma (*n* = ≤5); EBV+ DLBCL (*n* = 6); gray zone lymphoma/B-cell lymphoma, unclassifiable (*n* = 6); primary cutaneous DLBCL, leg type (*n* = ≤5), and T-cell/histiocyte-rich LBCL (*n* = ≤5).

^e^Patients who did not achieve a partial or complete response to first-line therapy and patients with documented progression within 6 months from the last known treatment during the first line.

^f^Patients who achieved a partial or complete response to first-line therapy and documented progression 6–12 months from the last known treatment during the first line.

^g^Number of patients who had next line of therapy recorded in the database.

^h^Number of patients with documented evidence for use of R-GemOx as a bridge to CAR T-cell therapy.

**TABLE S6.** **Subgroup analysis for R-GemOx real-world outcomes among patients with R/R LBCL.**

| **Efficacy parameter** | **All patients**  **(*N* = 281)** | **R-GemOx in the 2L**  **(*n* = 146)** | **R-GemOx in the 3L+**  **(*n* = 135)** | **Age ≥65 years**  **(*n* = 190)** | **Age ≥75 years**  **(*n* = 109)** | **R-GemOx not used as bridging therapy  *(n = 222)*** | **Primary  refractory disease  (*n* = 194)** | **DLBCL NOS**^a^  **(*n* = 178)** |
| --- | --- | --- | --- | --- | --- | --- | --- | --- |
| **ORR rate**,  % (*n/N*)  [95% CI] | 45.4 (84/185^b^)  [38.1–52.9] | 45.5  (45/99^c^)  [35.4–55.8] | 45.3  (39/86^c^)  [34.6–56.5] | 50.4  (64/127^c^)  [41.4–59.4] | 53.8  (42/78^c^)  [42.2–65.2] | 45.5  (66/145^c^)  [37.2–54.0] | 38.1  (48/126^c^)  [29.6–47.2] | 47.5 (57/120^c^)  [38.3–56.8] |
| **CR rate**,  % (*n/N*) [95% CI] | 22.2 (41/185^b^)  [16.4–28.8] | 26.3  (26/99^c^)  [17.9–36.1] | 17.4  (15/86^c^)  [10.1–27.1] | 22.0  (28/127^c^)  [15.2–30.3] | 21.8  (17/78^c^)  [13.2–32.6] | 17.9  (26/145^c^)  [12.1–25.2] | 15.9  (20/126^c^)  [10.0–23.4] | 25.0  (30/120^c^)  [17.5–33.7] |
| **Median PFS**^d^, months  [95% CI] | 2.8 [2.6–3.7] | 3.5 [2.6–4.9] | 2.7 [2.2–3.6] | 3.0  [2.5–4.5] | 3.5  [2.6–5.1] | 2.8  [2.2–3.5] | 2.7 [2.2–3.5] | 3.2  [2.3–4.4] |
| **Median EFS**^d^, months  [95% CI] | 2.3  [2.1–2.8] | 2.6 [2.1–3.8] | 2.2 [1.8–2.7] | 2.5  [2.1–3.4] | 2.7  [2.1–4.1] | 2.3  [2.1–2.8] | 2.2 [2.0–2.7] | 2.2  [2.0–3.0] |
| **Median TTNT**^e^, months  [95% CI] | 3.9  [3.4–4.8] | 3.8 [3.0–4.9] | 3.9 [3.5–5.2] | 4.4  [3.7–5.2] | 4.6  [3.2–6.0] | 3.9  [3.4–4.8] | 3.7 [2.8–4.6] | 3.9  [3.2–5.0] |
| **Median OS**^e^, months  [95% CI] | 12.7  [10.6–17.1] | 19.6 [13.5–53.8] | 10.0 [7.9–12.7] | 12.4  [10.0–18.5] | 13.5  [9.7–25.3] | 10.6  [7.9–13.1] | 10.1 [8.5–12.5] | 16.1  [12.4–24.8] |

Abbreviations: 2L, second line; 3L+, third line or later; CI, confidence interval; CR, complete response; EFS, event-free survival; DLBCL, diffuse large B-cell lymphoma; LBCL, large B-cell lymphoma; NOS, not otherwise specified; ORR, overall response rate; OS, overall survival; PFS, progression-free survival; R-GemOx, rituximab with gemcitabine and oxaliplatin; R/R, relapsed or refractory; TTNT, time to next treatment.

^a^Patients with transformed disease and double/triple-hit status were excluded.

^b^Conducted in response-evaluable patients with ≥6 months of follow-up.

^c^Conducted in all response-evaluable patients with any length of follow-up.

**^d^**Analysis was conducted in all patients, apart from three who were not assessed for progression.

**^e^**Analysis was conducted in all patients.

**FIGURE S1. Flow chart of patients included in main analysis and sensitivity analyses of response outcomes.**

Abbreviations: EHR, electronic health records; R-GemOx, rituximab with gemcitabine and oxaliplatin. **FIGURE S2. Utilisation of (A) 2L and (B) 3L+ treatments for patients with R/R DLBCL from 2011 to 2024.**

Abbreviations: 2L, second line; 3L, third line; 3L+, third line or later; BR, bendamustine and rituximab; CAR T, chimeric antigen receptor T-cell; DLBCL, diffuse large B-cell lymphoma; pola-BR; polatuzumab vedotin, bendamustine, and rituximab; R-GemOx, rituximab, gemcitabine, and oxaliplatin; R/R, relapsed or refractory; Tafa-Len, tafasitamab plus lenalidomide.

**FIGURE S3. Duration between cycles of R-GemOx.**

Patients with available data: *n* = 237.

Excludes long tail on the right beyond 6 weeks with low frequencies.

Abbreviations: R-GemOx, rituximab with gemcitabine and oxaliplatin.
